# Towards specialised and differentiated long-term care services: a cross-sectional study

**DOI:** 10.1186/s12913-020-05647-y

**Published:** 2020-08-26

**Authors:** Hanne Marie Rostad, Marianne Sundlisæter Skinner, Ragnhild Hellesø, Maren Kristine Raknes Sogstad

**Affiliations:** Centre for Care Research, Norwegian University of Science and Technology (NTNU), Gjøvik, Norway

**Keywords:** Health services research, Home care services, Long-term care, Nursing homes, Primary health care, Service provision, Specialisation, Survey

## Abstract

**Background:**

Numerous forces drive the evolution and need for transformation of long-term care services. During the previous decade, primary health care has assumed increased responsibility for developing and providing care services, but there is still limited knowledge about how European care service systems are evolving to address new tasks and patients. Based on data from Norwegian municipalities, this study aims to (1) describe the availability of specialised services in Norwegian nursing homes and home care services and (2) analyse whether structural factors, like population size and/or centrality, are associated with the availability of specialised services in nursing homes and home care.

**Methods:**

This is a cross-sectional study of survey data. An online survey was designed specifically for this study. Its questions were developed from a comprehensive review of the literature and in partnership with a user panel. One representative from all of Norway’s 422 municipalities were invited to answer the survey from February to April 2019. In total, 277 municipalities completed the survey (response rate 66%). Chi-square analysis and Fisher’s exact test were used to test the associations between different categorical variables.

**Results:**

Specialised care services were highly prevalent. For example, there were nursing home units specialising in dementia care (89%) and rehabilitation (81%) and home care teams for dementia care (79%) and reablement (76%). Approximately two-thirds of our sample were categorised as having high availability of specialisation in nursing home and home care services. The larger, more central municipalities had higher availability of specialisation compared to medium-sized and small, less central municipalities.

**Conclusions:**

Our study indicates that a majority of nursing homes and home care services provide specialised and differentiated services that serve patient groups of different ages and diagnoses. Municipalities’ population size and centrality are associated with availability of specialised services in nursing homes and home care services.

## Background

Decision-makers in Europe argue that the status quo of primary health care cannot be maintained [1, 2] due to the aging of the population, the increasing number of patients with complex and multiple long-term conditions, the move from secondary care to primary care and limited growth in the primary care workforce [2]. Further, emphasis on quality and personal choice, technological innovation, financial pressure to limit public costs and political and ideological values (e.g. people should live in their own homes as long as possible rather than receive care in an institution) is driving the evolution and need for transformation in primary health care [1, 3–5].

Before we position our study within the scientific field, we must clarify some key terms in this paper – ‘primary health care’ and ‘long-term care’ – as health care systems and terminology vary across countries. ‘Primary health care’ is understood as a broad term that covers health care services throughout one’s lifespan, ranging from promotion and prevention to treatment, rehabilitation and palliative care [6]. These services are provided by different professionals in different settings. ‘Long-term care’ is part of primary health care, and it involves services specifically directed at people who cannot care for themselves over a period of time due to, for example, chronic illness or disability. It involves a variety of services provided by different caregivers to address medical and non-medical needs, which are provided at home, in assisted living facilities or in nursing homes [7].

The organisation of primary health care varies between countries, even among countries with social and cultural similarities and similar health and funding systems. Still, in many European countries, one of the key policy issues during the previous decade has been the reorganisation of primary health care, including long-term care, and introduction of new organisational models [8, 9].

As in other European countries, long-term care in Norway has assumed increased responsibility for developing and providing care services [10, 11]. Patients are discharged from hospitals (i.e. secondary care) to primary health care quicker [12, 13] and sicker than before [14]. In addition, the aging population with chronic conditions and multi-morbidity increases the demand for long-term care for older adults. All these factors have resulted in new tasks and new patient groups in primary health care, increasing the demand for the development of services with appropriate structures, content and competences [15].

We have limited knowledge about how European care service systems are evolving to address these new tasks and patients. Which types of services and availability of specialisation are offered by providers of long-term care? And do local governments (such as counties, municipalities and districts) with different characteristics deal with these new tasks and patients differently? The findings of a qualitative study conducted in selected Norwegian municipalities in 2014 indicated that nursing homes served a broader set of individuals compared to 10 years ago, when nursing homes primarily provided general services to older adults [16]. Now, people in need of palliative care or people with substance abuse issues may receive care in nursing homes, although this differentiated form of nursing home care seems to be provided primarily in large municipalities [17]. However, there is still a knowledge gap concerning whether and how local care service models are emerging in the long-term care service landscape following the introduction of new patients and tasks. This study represents an original contribution to the knowledge by using new data on Norwegian long-term care to [1] describe the availability of specialised services of nursing homes and home care services and [2] analyse whether structural factors, like population size and/or centrality, are associated with the availability of specialised services in nursing homes and home care.

Research in this area is essential because improved knowledge and understanding of current services models aids both national and international policymakers who plan, set priorities and develop services for the future, as well as local stakeholders who need information about which types of services are provided to their country’s or region’s inhabitants.

## Methods

### Setting and participants

This cross-sectional study analyses survey data from long-term care settings (i.e. nursing homes and home care services) in Norway. When we conducted our study in 2019, Norway had 422 municipalities, but this number is declining due to ongoing municipal reform [18].

Municipalities are legally responsible for the provision of primary health care services, such as general practitioner services, as well as rehabilitation and long-term care services, like home care and nursing home services. Home care services in Norway provide help, assistance, health care and treatment for older adults and people who are sick or have disabilities and are living at home. Nursing home services can be separated into short-term and long-term stays. Short-term nursing home stays are time-limited, lasting from one day to 12 weeks and are often provided after hospitalisation or as respite care. The goals of short-term stays are to assess the person’s needs regarding help, assistance and guidance and to enable the person to return home or postpone institutionalisation. In contrast, long-term nursing home stays are for people – primarily older adults – with extensive medical and care needs who are unable to live at home as they require full-time care [19].

Primary health care in Norway, including long-term care, is mainly financed by municipalities’ so-called ‘unrestricted revenues per capita’ (tax revenues and government grants) as well as fees and user payments. Municipalities have some autonomy in how they spend their unrestricted revenues per capita, but they are bound by the requirements for service content and quality, stipulated in Norwegian law.

All of Norway’s 422 municipalities were invited to answer the survey via email. First, an email was sent to the municipalities’ email reception, which handles all inquiries, requesting the contact information of a person who could respond to the survey on behalf of the municipality. There were no specific eligibility criteria, but we emphasised that the respondent needed to be knowledgeable about the organisation of the municipality’s long-term care services. The titles of the respondents varied due to variation in the municipalities’ organisational structures. The person designated as the respondent was contacted via email, which provided information about the study and participation in addition to a link to the survey. Email reminders were sent three times during the study period, approximately every four weeks.

### Data sources

Data were collected over a three-month period, from February to April 2019. An online survey was designed specifically for this study. The questions were developed from a comprehensive review of literature, including research [17, 20], reports [15, 16] and white papers [21, 22], and in partnership with a user panel. The panel consisted of representatives from five municipalities of different sizes (based on population) and geographical locations. The representatives held different positions as leaders and advisers at different organisational levels, but all working in or for long-term care services. The research team arranged two face-to-face meetings to discuss the development of the survey and consulted with members of the panel via e-mail between and after meetings.

Norwegian municipalities vary significantly in terms of size, topography and population composition, and so it was necessary to have back-and-forth discussions with the panel in order to develop a survey that would be perceived as valid across municipal differences. The survey was reviewed twice by the user panel and piloted by three representatives from the target group. Adjustments were made based on feedback from the user panel and pilot. Specifically, questions were re-phrased or removed due to lack of relevance.

The goal of the survey was to map the municipality’s provision of long-term care services for adults (i.e. individuals over the age of 18). The survey consisted of 40 possible questions. As we used conditional branching, the respondent’s path through the survey varied based on their answers. The survey questions analysed in this paper concerned services provided in nursing home and home care services specifically (Table 1). It was possible for respondents to provide additional information via text boxes. Some of the text box answers are used as supplementary material in the discussion.

**Table 1:** Survey questions

**Long-term care in nursing homes**	
• Does your municipality provide long-term care in nursing homes? (yes/no). If ‘yes’, the next question would appear.	
• Some municipalities have specialised long-term care services for different patient groups. Please check the box if your municipality has long-term care in nursing homes for the following (choosing multiple options is possible): Dementia care Reinforced dementia care Neurological disorders Psychiatric disorders Substance abuse None of these	
**Short-term care in nursing homes**	
• Does your municipality provide short-term care in nursing homes? (yes/no). If ‘yes’, the next question would appear.	
• Some municipalities have specialised short-term care services for different patient groups. Please check the box if your municipality has long-term care in nursing homes for the following (choosing multiple options is possible): Rehabilitation Dementia care Palliative care Neurological disorders Psychiatric disorders Medical intermediate care Substance abuse None of these	
**Specialised teams in home care**	
• Please check the box if your municipality has teams/groups (multiple people working together to achieve a common goal) for the following services in home care (choosing multiple options is possible): Reablement Palliative care Dementia care Oncological care Substance abuse Psychiatric care Telecare None of these	

### Quantitative variables

The variables collected through the survey are dichotomous or categorical (nominal).

To describe the availability of specialised services, we computed variables based on the number of specialised services provided: one variable for nursing home and one for home care. Initially, these were discrete variables which were then categorised to achieve more intuitive interpretation and better understanding of the relationship between this study’s variables. For nursing homes, the categorisation of availability of specialised services is based on the number of different patient groups to whom specialised services are provided in the municipality’s nursing home(s): ‘no’ (0 patient groups), ‘low availability’ (1 or 2 different patient groups) and ‘high availability’ (3 or more different patient groups). For home care, specialisation is based on the number of specialised teams established within the municipality’s home care service(s): ‘no’ (0 teams), ‘low availability’ (1 or 2 different teams) and ‘high availability’ (3 or more different teams). We chose to categorise the availability of specialised services in this way based on discussion among the research team, previous research on the topic of specialisation in long-term care [16, 17] and what seems sensible and reasonable from a practice perspective. Fit for the data was also taken into consideration where we explored the frequency distribution to decide how to group the data into the appropriate categories.

We obtained data on the municipalities’ *population size* and *centrality* from publicly available statistics (Statistics Norway).

#### Population size

We applied the classification used by Statistics Norway, which includes three categories [23]: small (< 4999 inhabitants), medium (5000–19,999) and large (≥ 20,000 inhabitants). The data are from the first quarter of 2019.

#### Centrality

Statistics Norway calculates a centrality index based on travel time to workplaces and places that provide goods and services [24]. We used three categories of centrality: least central, central and most central. The data are from January 2018.

### Statistical methods

Data were analysed using SPSS version 25. Differences between respondents and non-respondents in terms of population size and centrality were cross-tabulated and tested for associations by chi-square analysis. Fisher’s exact test was used to test the association between population size and/or centrality and availability of specialised services. Frequencies were used to describe the distribution of nursing home services and home care teams within the municipalities. There were no missing values.

## Results

In total, we obtained complete responses from 277 municipalities. The response rate was 66%. We compared respondents and non-respondents in terms of population size and centrality. It seems that the smallest municipalities (in terms of population size) were somewhat underrepresented in our sample, whilst the medium-sized were slightly overrepresented (Table 2).

**Table 2:** Comparison of respondents and non-respondents, n (%)

	Respondents *n* = 277	Non-respondents *n* = 145	*p*-value
Population size			0.03
≤ 4999	131 (47)	88 (61)	
5000–19,999	105 (38)	39 (27)	
≥ 20,000	41 (15)	18 (12)	
Centrality			0.20
Group 1 (most central)	23 (7)	7 (5)	
Group 2	106 (37)	49 (34)	
Group 3 (least central)	148 (56)	89 (61)	

In the following, we present the findings regarding nursing homes, followed by the findings regarding home care services.

### Nursing homes

Nearly all the municipalities (98%) provided long- and short-term services in nursing homes (Table 3). Specialised dementia care was highly prevalent in long-term nursing home stays, whilst rehabilitation and palliative care were the most prevalent types of specialised services during short-term stays. Specialised nursing home services for neurological disorders, psychiatry and substance abuse were less widespread.

**Table 3:** Provision and specialisation of nursing home services, n (%)

Specialised services for	Long-term 273 (98)	Short-term 272 (98)
Rehabilitation	N/A	224 (81)
Palliative care	N/A	194 (70)
Medical intermediate care ^a^	N/A	139 (50)
Dementia care	247 (89)	97 (35)
Reinforced dementia care ^b^	91 (33)	N/A
Psychiatric disorders	35 (13)	51 (18)
Neurological disorders	8 (3)	31 (11)
Substance abuse	15 (5)	29 (10)

N/A (not applicable) means that the specialised service was not one of the multiple-choice options in the survey (e.g. we did not ask about ‘rehabilitation’ for long-term care stays).

^a^ Medical intermediate care is a nursing home unit for people in need of medical care or follow-up after discharge from hospital

^b^ Reinforced dementia care is a nursing home unit for residents with dementia and additional behavioural and psychological symptoms

Most municipalities had one or more nursing home(s) with specialised long- and short-term stays. For short-term stays, 57% of the municipalities had three or more different types of specialised services while only 12% did for long-term nursing home stays (*p* < 0.01) (Table 4).

**Table 4:** Availability of specialised services in nursing home services measured as the number of services targeted at different patient groups, n (%)

	Long-term (*n* = 273)	Short-term (*n* = 272)	*p*-value
Specialisation			< 0.01
No (0)	23 (8)	33 (12)	
Low [1, 2]	219 (80)	85 (31)	
High (more than 3)	31 (12)	155 (57)	

We found a statistically significant association between population size, centrality and availability of specialised services in both long- and short-term nursing home services (Table 5). The large and more central municipalities were more likely to have high availability of specialised services compared to the medium-sized and small municipalities.

**Table 5:** Impact of population size and centrality on the availability of specialised services in nursing home services

	Long-term				Short-term			
	No specialisation	Low availability of specialised services	High availability of specialised services	*p*-value	No specialisation	Low availability of specialised services	High availability of specialised services	*p*-value
Population size				< 0.01				< 0.01
≤ 4999	16 (12)	111 (86)	3 (2)		24 (19)	49 (31)	66 (50)	
5000–19,999	7 (7)	89 (86)	7 (7)		8 (8)	38 (37)	57 (55)	
≥ 20,000	0 (0)	19 (48)	21 (52)		1 (3)	7 (18)	32 (79)	
Centrality				< 0.01				0.01
Most central	1 (4)	12 (52)	10 (44)		2 (9)	4 (17)	17 (74)	
Central	2 (2)	83 (81)	18 (17)		8 (8)	44 (43)	51 (49)	
Least central	20 (14)	124 (84)	3 (2)		23 (16)	37 (25)	87 (59)	

### Home care services

Specialised home care teams for dementia care and reablement were most prevalent, appearing in over three quarters of our sample (79 and 75% respectively). Specialised teams for palliative care, oncological care, substance abuse, psychiatric care and telecare were available in approximately half of the municipalities (Table 6).

**Table 6:** Specialisation of home care services (*n* = 277)

Specialised teams for	n (%)
Dementia care	218 (79)
Reablement	210 (76)
Psychiatric care	162 (59)
Oncological care	149 (54)
Telecare	145 (52)
Substance abuse	142 (51)
Palliative care	129 (47)

Only nine of the 277 municipalities reported having no specialised teams for home care, while 69% were categorised as municipalities with a high availability of specialised services (Table 7).

**Table 7:** Availability of specialised services in home care measured as the number of different types of specialised teams, n (%)

	(*n* = 277)
Specialisation	
No (0)	9 (3)
Low [1, 2]	61 (28)
High [3–7]	207 (69)

The association between population size and specialisation in home care services was statistically significant, but the association with centrality was not (Table 8). A significantly higher number of the larger and medium-sized municipalities had high availability of specialised services compared to small municipalities.

**Table 8:** Impact of population size and centrality on the availability of specialised services in home care services

	Home care services			
	No specialisation	Low availability of specialised services	High availability of specialised services	*p*-value
Population size				0.03
≤ 4999	7 (5)	37 (28)	87 (67)	
5000–19,999	1 (1)	17 (16)	87 (83)	
≥ 20,000	1 (2)	7 (17)	33 (81)	
Centrality				0.13
Most central	0 (0)	4 (17)	19 (83)	
Central	1 (1)	19 (18)	86 (81)	
Least central	8 (5)	38 (26)	102 (69)	

## Discussion

The aims of this study are to describe the availability of specialised services of Norwegian long-term care services and analyse whether population size and/or centrality are associated with the availability of specialised services. The key findings are that the majority of nursing homes and home care provide differentiated and specialised services, and that availability of specialised services is associated with population size and centrality.

In terms of nursing home services, both long- and short-term stays were provided to a multitude of patient groups. Specialised care for people with dementia was most prevalent services provided during long-term nursing home stays, while rehabilitation and palliative care were the two most prevalent services provided during short-term stays. These findings are in line with those of previous studies [17, 25]. Home care was also provided by specialised teams for several different patient groups, most commonly for those requiring dementia care and reablement.

Traditionally, long-term care services in Norway have adopted a generalist approach, meaning they were intended to serve a broad group of patients, primarily older adults over the age of 67 [16]. Our data and previous research [16, 17] suggest that there has been a considerable shift in the care service landscape during the last 10–20 years, and new care service models include a wide range of specialised services. Increased awareness of the care needs of patients with complex and multiple long-term conditions, who are prevalent recipients of long-term care, could be one explanation for the increase in targeted and specialised health and social care services.

The availability of specialised services varied between municipalities; small and rural municipalities often had lower availability of specialised services while large and central municipalities, often had higher availability of specialised services. Still, municipalities are required by law to provide competent and high-quality healthcare services to all inhabitants who need it, but due to differences in size, topography and demography, they must meet very different needs when providing care to their inhabitants. We found that long-term care services in larger, more central municipalities are, to a greater extent, characterised by higher availability of specialised services compared to smaller, less central municipalities. Thus, we identified different service models; some municipalities provide more specialised services, while others are more generalists in their approach. This is in accordance with the findings of previous studies [16, 17]. However, our study cannot show that small and/or less central municipalities do not provide the specialised services in question or that large and/or central municipalities do not provide, or have impaired capacity for, generalist services. This can be illustrated by comments made during the survey by respondents from small municipalities, where these municipalities seem to aim for flexibility in their service provision and reallocate or redistribute their services according to changing needs. Specifically, respondents commented that nursing home beds were used in a flexible way, that everyone was provided with the services they needed, even if there were no earmarked beds, and that staffing levels were adapted according to needs.

Different models for service provision across municipalities may be justifiable for several reasons in a country like Norway. Factors such as centrality, population size, demographics and the population’s health and care needs, naturally create different service models. Small, less central municipalities are likely to have more limited opportunities to develop and maintain specialised and differentiated services, as their populations are smaller and often spread out over large geographical areas. Larger, more central, municipalities, on the other hand, have larger volumes of patients in different target groups, meaning that it is sustainable to offer more specialised services for a variety of patient groups. In addition, local needs-driven innovations may necessarily lead to variation [26].

We have identified that the availability of specialised long-term care services contributes to different services models. There is no ‘best’ service model or a model that would fit all. However, the identification and characterisation of different service models is a necessary first step in order to improve the quality, efficiency and sustainability of long-term care [20] as information about what types of service models emerge from the availability of specialised services is necessary for subsequent analyses of how these models may influence outcomes on the micro, meso and macro level. To develop knowledge about outcomes associated with different services models is an important avenue for further research as the availability of specialised services can impact on the quality of care [20].

Availability of specialised services in long-term care are both advantageous and necessary. For example, a previous study indicated that dedicated palliative teams in home care were associated with improvement in nausea, anxiety, easier access to staff, global quality of life in patients, and more [27]. Furthermore, a study comparing quality of life scores in dementia care units with traditional units in nursing homes found that the specialised dementia care units scored significantly higher on a number of indicators [28]. On the other hand, specialisation may have some unintended consequences for what services are available, to whom and of what quality.

Since specialisation reflects prioritisation and can bring about changes in prioritisation for different patient groups and types of services, concerns have been raised about how specialisation and differentiation of services may affect the access, comprehensiveness and integration of care for older adults [29]. More patients and more diversified patient groups now compete for the same amount of resources, and many older adults risk falling outside the criteria for receiving specialised treatment [29]. Additionally, many people receiving long-term care services have several comorbidities and thus have complex needs. In that regard, whether availability of specialised services may give rise to fragmentation of services based on individual diagnoses rather than an integrated and comprehensive care approach [17], needs to be investigated. This may lead to challenges regarding the coordination of care for service providers, patients and their next-of-kin because different needs will be addressed by different providers.

The emergence of different services models by the availability of specialised service is a natural result of new patient groups and more tasks entering long-term care. Having specialised service places higher demands on staff to develop competencies in order to ensure high-quality care. Thus, it is worrying that previous research has found that the level of competence in community elderly care is insufficient in several areas and that there is a general lack of opportunities for competence development [30, 31]. An investigation conducted by the Office of the Auditor General concluded that there has only been a low level of competence development since the Norwegian primary health care sector was given responsibility for new and more tasks [32]. Many countries are facing difficulties recruiting and keeping expertise in care settings dominated by older patients [33], such as nursing homes [34]. Thus, if the necessary expertise is lacking, the full potential of specialised long-term care services may not be fulfilled.

Furthermore, it is crucial for future research to investigate whether different models of long-term service provision threaten horizontal equity (i.e. equal treatment of individuals or groups in the same circumstances) and leads to variation in the quality of care. I addition, studies should examine how the availability of specialised services can affect the scope of innovative approaches and solutions. Tingvold and Magnussen (2018) raise the unanswered question whether municipalities providing services with a more generalist approach are better at, for example, individualising care. Having no or few permanent specialised services means that when a specialised service is needed, the municipality must assemble it based on individual needs in that given situation. Additionally, one could also question how structures of care that are likely to affect quality (e.g. facilities, equipment, staffing levels) are maintained when a service is not provided and practiced on a regular basis.

Overall, our study indicates that different service models have emerged following the availability of specialised services in long-term care. This knowledge and understanding can allow the municipalities responsible for service delivery to use this knowledge to reflect on their chosen model of service delivery, which could be more or less intentional, and plan for future services. Furthermore, policymakers can use this knowledge to continuously develop and improve targeted and successful policies by analysing whether these different services models are associated with patients’ health and services outcomes – a question that is also important for patients and their families.

### Strengths and limitations

The study is based on municipal managerial employees’ knowledge and perception of the provision of long-term care services, not observation or reports by the service providers themselves. The respondents may not have had complete knowledge about the full extent of long-term care activities since they were not engaged in hands-on work in the field, although some respondents are likely to have been closer to the practice field than others.

Due to differences in the organisation of these services between municipalities, we chose to leave it up to the municipalities to decide who would be most qualified to answer the survey based on their organisational structure. This resulted in variation in the titles held by the respondents. We do not consider this variability to be a weakness of the study, nor do we believe it has impacted the findings, since the person’s knowledge of the organisation of care services is more related to their function, task and responsibilities than to title within the municipality.

Surveys do not generate in-depth knowledge. Our questions were standardised, and we were only able to ask general questions in order to ensure they were relevant to a broad range of municipalities. Because of this, our survey results may not be as valid as if we had obtained results using other methods of data collection that allow the researcher to more comprehensively examine the topic of study. However, we consider it a strength that respondents were able to elaborate on their responses in the text boxes.

Another limitation is that we cannot provide insight into how long-term care services are organised and delivered in different municipalities or how they impact on dimensions of service quality. However, our study has been designed to facilitate long-term follow-up of the development of primary health care in Norway in order to gain more detailed knowledge about how international and national trends and reforms change the care landscape.

The categorisation of our variable ‘availability of specialised services ‘was based on findings from previous research, our knowledge of the field and the fit of the variable with our data. Therefore, a different categorisation of ‘availability of specialised services’ would provide different estimates.

The sample in our study covers 66% of the population, but small municipalities (in terms of population size) seem somewhat underrepresented in our sample (Table 2). One reason for this might be that small municipalities did not perceive the questions as relevant to their model of service provision, since many dealt with specialised services for various patient groups. Other reasons might be that managers in smaller municipalities have more limited administrative resources and broader responsibilities and thus are more likely to decline participation in surveys due to time constraints and/or survey fatigue. However, we do not believe that the underrepresentation of smaller municipalities led us to draw a faulty conclusion, as our findings are in line with previous similar studies (although there are few, and they are methodically different) [16, 17].

The organisation of long-term care is quite different across countries, thus limiting the generalisability of the findings from this study. However, this study may provide policymakers and other stakeholders with some insight into how continuing political and social changes can impact long-term care services and demonstrate the need to uncover trends and discuss their intended effects and unintended consequences, both in individual countries and globally. This can help stakeholders plan for sustainable, high quality primary health care services in the future.

## Conclusion

The majority of nursing homes and home care services provide differentiated and specialised services which are likely to meet the needs of patients of different ages with a diverse diseases and diagnoses. Municipal characteristics, such as population size and centrality, are associated with the availability of specialised services in nursing home and home care services. Our data could not show whether different service models in long-term care impact on, for example, the quality or efficiency of the delivered care, but we argue why this is an important avenue for future research.

## Data Availability

The datasets used and/or analysed during the current study are available from the corresponding author on reasonable request.
